# A randomized clinical trial of an integrated behavioral self-management intervention Simultaneously Targeting Obesity and Pain: the STOP trial

**DOI:** 10.1186/1471-2458-14-621

**Published:** 2014-06-18

**Authors:** E Amy Janke, Megan Fritz, Christina Hopkins, Brittany Haltzman, Jessica M Sautter, Michelle L Ramirez

**Affiliations:** 1 Department of Behavioral and Social Sciences, University of the Sciences, 600 South 43rd Street, Philadelphia, PA 19104, USA

**Keywords:** Pain, Obesity, Pragmatic randomized clinical trial, Mixed methods

## Abstract

**Background:**

Obesity often occurs co-morbid with chronic, non-cancer pain. While behavioral treatments have proved effective for pain management and weight loss independently, integrated interventions are lacking. The study Simultaneously Targeting Obesity and Pain (STOP) is a prospective, pragmatic, randomized controlled trial that aims to determine whether overweight/obese individuals with chronic pain who are randomized to receive an integrated treatment Simultaneously Targeting Obesity and Pain (STOP) will show more weight loss and greater reduction in pain intensity over a 6-month period and greater maintenance at 12 months than those who receive standard care behavioral weight loss or standard care behavioral pain management. We hypothesize that individuals randomized to receive the STOP treatment will demonstrate improved weight loss, pain reduction, and maintenance compared to standard care treatment approaches.

**Methods/Design:**

Adults aged ≥ 18 with a body mass index ≥ 25 and who report persistent pain (≥4 out of 0–10 for > 6 months) will be recruited for treatment at the Health Behavior Research Lab at the University of the Sciences. After baseline assessments and goal setting, participants will be randomized to receive one of three treatments. Participants will receive eleven treatment sessions delivered during 1 hour, weekly individual meetings with a clinic therapist. Follow-up will occur at 3, 6 and 12-month time points; assessments will include measures of weight and pain intensity (primary outcomes). A mixed-method approach to evaluating study outcomes will include individual interviews with participants about their treatment experience. These interviews will be led by a research staffer who was not involved in study intervention or assessment using a semi-structured discussion guide.

**Discussion:**

This study fills an important gap in intervention research, evaluating best-practices for behavioral management of a highly prevalent co-morbidity that has sub-optimal outcomes with currently-implemented approaches. STOP’s pragmatic focus builds upon treatments already in use in clinical practice. Should STOP be found efficacious in achieving the dual outcomes of pain management and weight loss, such an approach could be integrated into practice with minimal additional cost or training.

**Trial registration:**

Clinical Trials.gov NCT02100995 Date of Registration: March 2014

## Integrated behavioral self-management simultaneously targeting obesity and pain: the STOP trial

### Background

Obesity continues to be a prevalent public health problem associated with high rates of morbidity and mortality [1-4]. Many of the conditions found co-morbid with obesity present with persistent pain [5], and evidence demonstrates a relationship between higher body mass index (BMI) and prevalence of chronic non-cancer pain [6]. Pain-related conditions associated with overweight are numerous and include rheumatoid arthritis, osteoarthritis, fibromyalgia, low back pain, headache, and neuropathic pain conditions [4,5,7]. While the economic impact of co-morbid pain and obesity is unknown, it is likely significant as separately chronic pain and obesity are prevalent and costly conditions associated with high levels of health care utilization [2,8,9]. Pathways linking obesity and pain are likely complex and multifactorial, including mechanical, structural, metabolic, and behavioral factors [10].

Behavioral treatments have demonstrated efficacy in achieving improved pain management and weight loss for individuals with chronic pain or overweight separately [11-13]. However, research examining optimal treatment approaches for individuals with co-morbid chronic pain and obesity is limited. Current evidence suggests that for individuals with overweight and osteoarthritis, weight loss achieved via exercise and dietary changes may improve pain outcomes [14-16]. Research has also shown a correlation between the amount of weight lost and degree of pain symptom improvement [17]. However, such findings are drawn from clinical trials delivering a highly-controlled weight loss intervention, typically caloric restriction and/or physical activity, in narrowly defined and selectively recruited populations, such as individuals with knee osteoarthritis. Interventions simultaneously targeting pain and weight symptoms in individuals with obesity and chronic pain have not been widely examined and treatment programs offered in most clinical settings often focus singularly on pain or weight concerns.

The lack of integrated interventions is unfortunate because practitioners and patients report pain as a barrier to weight loss and increased weight as a barrier to chronic pain self-management [18,19]. Furthermore, evidence suggests individuals presenting for behavioral treatment with co-morbid pain and obesity experience poorer weight loss [20] and pain management [21] outcomes when participating in evidence-based, behavioral programming encouraging self-management of pain symptoms or weight loss as a singular focus. Reasons for the attenuated outcomes are unclear, but qualitative data suggest individuals with chronic pain and obesity may struggle to engage in currently-available, empirically-based approaches to pain and weight management because these programs neglect to assess and accommodate for the unique challenges brought on by the comorbidity [22]. Furthermore, these individuals often observe synergies between their pain and weight symptoms and desire treatment options that would simultaneously address both, yet feel frustrated with a lack of available treatment options tailored to meet the needs of their comorbidity in an integrated fashion [23].

Encouragingly, evidence-based behavioral treatment approaches for both pain and weight management demonstrate overlap in treatment foci, such as an emphasis on physical activity, maintaining a healthy lifestyle, and behavioral components such as stress management and cognitive restructuring [12,13]. Therefore, it is possible to select and integrate shared components of each evidence-based program into a single program that simultaneously targets chronic pain and obesity. Creating an integrated program based on currently-available approaches to pain management and weight loss has several advantages. First, such a program would be based upon methods with demonstrated efficacy, increasing the likelihood for success. Second, an integrated treatment approach would likely mirror existing clinical practice more closely and, should such an approach be found efficacious, changes could be incorporated into existing programs more easily. Finally, emerging evidence suggests that programs simultaneously targeting pain [24] or obesity [25] alongside common co-morbidities may prove efficacious, and specifically targeting the co-occurrence of pain and obesity may result in improved pain, weight, and quality of life outcomes [26].

This paper describes the study protocol for the Simultaneously Targeting Obesity and Pain (STOP) study, a prospective, pragmatic, randomized controlled trial designed to compare the clinical effectiveness of three treatment approaches for the management of pain and obesity: 1) behavioral chronic pain management; 2) behavioral weight loss; and 3) and an integrated approach Simultaneously Targeting Obesity and Pain.

### Primary aim

The primary aims of the proposed research are to determine whether overweight and obese individuals with chronic pain who are randomized to receive an integrated treatment Simultaneously Targeting Obesity and Pain (STOP) will show more weight loss and greater reduction in pain intensity over a 6-month period and greater maintenance at 12 months than those who receive standard care behavioral weight loss (SCW) or standard care behavioral pain management (SCP). We hypothesize that individuals randomized to receive the STOP treatment will demonstrate improved weight loss, pain reduction, and maintenance compared to standard care treatment approaches.

### Secondary aims

Secondary aims are to determine whether overweight/obese patients with chronic pain who are randomized to the STOP treatment will show improved pain disability, improved quality of life, and greater treatment adherence compared to standard care treatments only. Ancillary outcomes that will be collected include mood and waist circumference. Processes of change will be examined using two approaches. Participants will complete assessments of eating behaviors, readiness to change, attitudes toward pain and self-efficacy for pain management, and therapy evaluation and preferences for treatment. Additionally, participants will be invited to participate in individual interviews assessing patient experience with treatment, preferences for treatment approaches, and priorities for treatment outcomes.

## Methods and design

### Ethical considerations

The Institutional Review Board (IRB) of the University of the Sciences (IRB# 288168–10) reviewed and approved all study materials including the study protocol, informed consent document, recruitment materials, and assessment instruments and approved the human research participant protection procedures for this trial. The IRB will approve all study protocol amendments and reportable adverse events throughout the trial. Research clinicians, study coordinators, laboratory staff and the Principal Investigator have completed the National Institutes of Health (NIH) online research ethics training course “Protecting Human Research Participants” and additional University of the Sciences training in the ethical practice of research. Written informed consent will be obtained from all study participants.

### Study design

**Figure 1:**
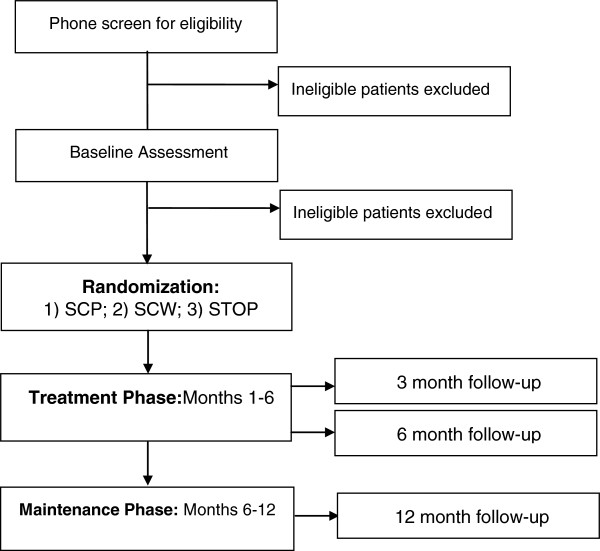
STOP trial phases.

The STOP trial is a prospective, pragmatic, randomized controlled trial. Pragmatic clinical trials focus on clinically-relevant outcomes important to patients and other relevant stakeholders, conduct research in settings similar to those in practice, include participants that resemble those seen in practice, and include real-world alternatives [27]. In the STOP Trial, we plan to enroll approximately 120 participants who report co-morbid chronic pain and overweight/obesity. Participants will be randomized to one of three parallel treatment groups: 1) standard care behavioral pain management (SCP), 2) standard care behavioral weight management (SCW), and 3) integrated behavioral self-management Simultaneously Targeting Obesity and Pain (STOP) (Figure 1).

### Research setting

Services will be provided within the Health, Eating, and Wellness (HEW) Clinic by members of the Health Behavior Research Lab (HBR Lab) at the University of the Sciences. In keeping with the pragmatic focus of the study, treatment will be provided by professionals with various clinical training including both Master’s and Doctoral-level trained therapists. The Health, Eating, and Wellness Clinic is a University-based, community-focused, training clinic co-Directed by the study principal investigator offering low-cost or free behavioral health services to members of the surrounding urban community.

### Study population

Participants will be recruited from the Philadelphia metropolitan area. A high degree of ethnic diversity characterizes this region. According to estimates by the United States Census Bureau, Philadelphia County is an ethnically diverse region of 1.6 million people with the following racial/ethnic composition: 45.7% White, 44.3%, Black, 13% Hispanic or Latino, and 6.8% Asian [28]. Racial/ethnic representation in the study sample is expected to reflect that in the surrounding community. Our target population will include adults of any age ≥ 18 who currently report chronic pain and are overweight or obese.

### Inclusion criteria

Adults aged ≥ 18 with body mass index (BMI) ≥ 25 and chronic pain will be recruited. To help differentiate between individuals with chronic pain and other, acute or transient pain complaints, we defined chronic pain as pain at a level ≥ 4 (on a scale of 0–10) on a majority of the days for 6 months or more prior to study participation. In keeping with the pragmatic study approach, chronic pain will be broadly defined to include any pain complaint that is not solely attributable to cancer. Thus, this may include musculoskeletal pain (e.g., osteoarthritis, fibromyalgia), pain associated with autoimmune disorders (e.g., rheumatoid arthritis), headache, or neuropathic pain. Individuals must also be interested in behavioral treatment for weight loss, interested in behavioral treatment for chronic pain, and able to communicate comfortably in English. Participants must be willing to accept randomization to one of the three treatment arms. However, all participants will be offered the chance to receive additional weight loss and/or pain management services of their choosing upon completion of the intervention phase.

### Exclusion criteria

In keeping with the pragmatic focus, exclusion criteria will be minimal and kept to those appropriate and necessary given the particular health needs of interested individuals and resources available in our clinic setting to meet these needs. Participants will be excluded and/or referred for other, more appropriate services if they (1) have an unstable medical or psychiatric condition (e.g., recent myocardial infarction, active suicidal ideation/behavior); (2) meet criteria for current substance abuse or dependence; (3) meet the criteria for bulimia; (4) are non-fluent in spoken or written English; (5) are pregnant, pregnant within the past 6 months, or trying to get pregnant in the next 3 months; (6) have significant cognitive or sensorimotor impairment precluding treatment engagement; (7) are already participating in a similar structured diet or exercise program or pain self-management program or plan to begin such a program outside the study during the next month; (8) are at risk for significant adverse cardiovascular events with moderate activity (e.g., has symptoms while walking, scheduled for stress testing within next 2 months); or (9) plan to relocate within the upcoming 12 months.

### Recruitment and screening procedures

Participants will be recruited from the local community directly by advertisement and by health provider referral. Advertisements will be placed in local publications, on local radio, and on local internet sites, including social networking sites. To increase minority participation, advertisements will be placed in local newspapers and at community gathering spots in the Southwest, West, and North Philadelphia neighborhoods. These communities are predominantly African-American and Hispanic in composition.

A brief telephone assessment will initially screen candidates for interest and appropriateness for study participation. A study staff member will explain the research, ascertain the individual’s interest in participating, and screen for entry and exclusion criteria. Individuals who, upon phone screening, appear to satisfy enrollment criteria will be scheduled for a baseline session that will include additional in-person assessment.

### Baseline and randomization

At the in-person baseline session and prior to randomization, the study will again be described to participants and the formal informed consent process will take place. Once consented, an intake assessment will be conducted and baseline assessments will be performed.

At the conclusion of the baseline assessment, study therapists will work with participants to set 3–5 goals for treatment. These goals are specific, behavioral targets for which the patient can reasonably expect and measure change during a 12-week period. Furthermore, these goals are guided by the patient’s interests and desired areas of change, regardless of the treatment to which they are assigned. These long-term goals will be broken down into weekly goals in therapy, collaboratively adopted for home practice, and monitored throughout the course of treatment in all three treatment arms. At the conclusion of the baseline session, the participant will be scheduled for their first treatment session during the upcoming 7 days.

Randomization will occur immediately prior to the first scheduled treatment session. Participants will be randomly assigned to one of three groups: behavioral weight loss (Standard Care Weight, SCW), behavioral chronic pain management (Standard Care Pain, SCP), or integrated behavioral treatment Simultaneously Targeting Obesity and Pain (STOP).

### Treatment approaches

#### Intervention sessions

Each of the three treatments will follow a similar intervention timeline, although intervention content will vary by treatment (see Table 1). Each intervention will consist of 11 sessions delivered during 1 hour, weekly individual meetings with a clinic therapist. Intervention content is based upon empirically-validated behavioral techniques for self-management of weight and chronic pain. These techniques are widely used in clinical practice and considered the ‘gold standard’ of treatment for behavioral weight loss and/or pain management [11-13]. Treatment manuals have been designed describing each of these three treatments in order to assure therapists are utilizing these evidence-based approaches. Furthermore, each session will be recorded and monitored for fidelity to evidence-based approaches and weekly meetings will take place to supervise study staff and discuss the appropriate application of treatment approaches for each participant.

**Table 1:** Intervention content by treatment arm

	**Standard Care Weight Loss (SCW)**	**Standard Care Pain Management (SCP)**	**STOP**
0	Baseline Goals Setting		
1	Psychoeducation about Weight; Introduction to Calorie Restriction and Self-Monitoring	Psychoeducation about Pain and Theories about Pain	Psychoeducation about Pain and Weight, Self-monitoring
2	Review of Calorie Counting, Nutrition Information, and Self-Monitoring	Pleasant Activity Scheduling	Review of Calorie Counting, Nutrition Information, and Self-monitoring
3	Stimulus Control and Dietary Behaviors	Understanding Automatic Thoughts and Health, Identifying Automatic Thoughts	Activities Reframed: Pleasant Activities and Physical Activity
4	Guiding Thoughts, Motivation, and Problem Solving	Evaluating and Challenging Automatic Thoughts	Time-based Activity Pacing
5	Physical Activity	Cognitive Restructuring: Identifying ‘Shoulds’ and Core Beliefs	Stimulus Control, Dietary Behaviors
6	Reviewing Weight and Primary Goals	Cognitive Restructuring: Identifying and Challenging Core Beliefs	Thoughts and Health: Identifying Automatic Thoughts and Cognitive Errors
7	Identifying and Modifying Automatic Thoughts	Relaxation Techniques	Evaluating and Challenging Automatic Thoughts
8	Evaluating and Challenging Automatic Thoughts	Time-based Activity Pacing	Cognitive Restructuring: Identifying and Challenging “Should” Beliefs
9	Targeting Thoughts, Feelings, and Behaviors that Influence Diet and Activity	Stress Management and Coping Self-Statements	Cognitive Restructuring: Identifying and Challenging Core Beliefs
10	Relaxation Techniques	Assertiveness, Anger Management, Communication	Relaxation Techniques
11	Review and Planning for Weight Maintenance	Review, Relapse Prevention, Flare Up Planning	Review, Weight Maintenance, Relapse Prevention, Flare Up Planning

Each session will begin with a review of assigned home practice and between-session work towards patient-defined goals for the past week, and end with the assignment of home practice and patient-defined goals for the upcoming week. Patients in the SCW and STOP treatment arms will review food diaries with therapists at the beginning of each session to assess progress with their logging of dietary intake and meeting assigned calorie goals. Patients in the SCW and STOP treatment arms will have weekly weigh-ins prior to their treatment sessions; patients in the SCP arm will be weighed weekly after their treatment session. Patients in the SCP and STOP treatment arms will complete a weekly three-item rating of pain intensity and pain interference prior to treatment sessions; patients in the SCW arm will complete this pain assessment after their treatment session.

Participants in the SCW and STOP treatments will be assigned daily calorie goals at their first treatment session. Caloric targets will be established using the Harris-Benedict equation [29] to calculate an individual’s basal metabolic rate and daily calorie requirements, assuming a sedentary activity level and subtracting 500 calories from this calculated figure. If, after meeting their calorie goals daily for 2 consecutive weeks, participants do not lose weight, they will be instructed to reduce their caloric intake in 200 kcal increments until they reach a calorie intake that yields a weight loss rate of approximately .5 to 1% of their current weight each week. No patient will be given an intake goal below 1200 kcal per day. Conversely, if weight loss occurs at > 3 pounds/week for 3 consecutive weeks, the calorie intake goal will be increased in 100 kcal increments until weekly weight loss goals are achieved for 2 consecutive weeks.

### Standard care weight loss intervention

The weight loss intervention includes content focused around nutrition and eating habits, stimulus control and behavioral change, and physical activity. This content has been chosen because of its demonstrated effectiveness and importance in behavioral interventions to reduce weight [11,12]. The first session is focused on psychoeducation and provides treatment rationale and an introduction to self-monitoring and calorie restriction. During the first session, a daily calorie goal is assigned. Participants are taught to use logs to track caloric intake and plan their daily dietary needs. Self-monitoring is reviewed as needed during session two, as is information about portion sizes and basic nutrition (i.e., how to read a nutrition label). Session three offers an introduction to stimulus control tailored to the participant’s needs. Triggers to eating are identified and strategies to address these triggers are reviewed. Session four reviews the participant’s motivation for weight loss, decisional balance around the pros and cons of weight loss, and problem solving obstacles to weight loss. The fifth session covers physical activity, with a focus on pleasant activities and small, daily changes in activity levels. During session six, the therapist and patient review the patient’s primary goals relevant to weight loss, activity, and dietary intake. Session seven and eight teach an introduction to cognitive restructuring with a focus on identifying, evaluating, and challenging automatic thoughts related to eating, activity, and weight loss. Session nine reviews distress tolerance and approaches to problem solving intense emotions/experiences related to eating. Session 10 reviews the impact of stress on health, the importance of regular relaxation practice, and stress management. Diaphragmatic breathing and a progressive muscle relaxation activity are taught at this session. Finally, treatment concludes with session 11 and a review of material covered and planning for ongoing weight loss.

### Standard care chronic pain intervention

The chronic pain intervention is focused around reconceptualizing pain, decreasing catastrophizing, and increasing self-efficacy for pain. This content has been chosen because of its demonstrated effectiveness and importance in non-pharmacological interventions to improve pain management [13]. The first session focuses on providing a treatment rationale and psychoeducation about pain and theories of pain (gate control theory vs. specificity theory). Session two discusses pleasant activity scheduling. As an introduction to cognitive restructuring, sessions three and four address the relationship between thoughts and health, and teach identifying, evaluating, and challenging automatic thoughts related to pain. Sessions five and six further address cognitive restructuring by teaching the participant to identify and challenge intermediate and core beliefs related to the experience of pain. During session seven, the relationship between stress and pain is further reviewed and diaphragmatic breathing and progressive muscle relaxation are taught. Session eight discusses time-based activity pacing. Session nine focuses on stress management and cognitive control of pain. During session 10, participants and therapists discuss anger management and communication approaches with a focus on assertive communication techniques. Finally, session 11 is spent reviewing material learned and planning for ongoing use of skills and relapse prevention.

### STOP intervention

The STOP treatment includes content designed to simultaneously and explicitly target both chronic pain and obesity. Treatment components are drawn from evidence-based interventions for chronic pain and obesity separately [11-13] and then integrated to influence both outcomes. Treatment begins with education about the relationship between chronic pain and overweight/obesity and an introduction to self-monitoring. Participants are given a daily calorie goal, taught how to log daily caloric intake, and encouraged to do so daily. The second session reviews the participant’s experience with logging calories. At this session, basic nutrition information, including portion sizes and how to read a nutrition label, is also reviewed. The importance of regular activity is discussed during session three, and while physical activity is encouraged activities are reframed to encourage engagement with any pleasant activity. Session four presents time-based activity pacing. During session five, interventionists work with patients to identify triggers for eating, low activity, and pain and develop a strategy to manage these triggers. Sessions six and seven focus on identifying, evaluating, and challenging automatic thoughts about pain and weight. Sessions eight and nine focus on identifying and challenging intermediate and core beliefs related to pain and weight. Session 10 discusses the importance of relaxation and teaches diaphragmatic breathing and progressive muscle relaxation. Finally, learned material is reviewed during session 11 and a plan for relapse prevention, flare up planning, and ongoing weight loss is discussed.

### Quantitative data collection

Data will be collected at various time points beginning at baseline and continuing through 12-month follow-up on continuous and dichotomous variables using the measures listed in Table 2. Anthropomorphic measures of weight, height and waist circumference will be collected at each weekly assessment session, as will measures of pain intensity and interference. Participants will be paid $30 to offset travel costs at the 12-month follow-up to encourage retention.

**Table 2:** STOP table of measures

**Measures**	**Baseline**	**Week 1 & 11**	**Wks 2-10**	**3, 6, 12-month follow-up**
Health Behavior/Medical/Pain History Questionnaire	X			X
**Pain and eating behavior**				
West Haven Yale Multidimensional Pain Inventory (WHYMPI) [30]	X			X
Survey of Pain Attitudes (SOPA) [31]	X			X
Arthritis Self-Efficacy Scale (ASES) [32]	X			X
Pain Catastrophizing Scale (PCS) [33]	X			X
Dutch Eating Behavior Questionnaire (DEBQ) [34]	X			X
**Stage of change short forms**				
Exercise [35]	X			X
Weight [36]	X			X
Pain [37]	X			X
**Psychological measures**				
Quality of Life (SF-36) [38]	X			X
Primary Care Evaluation of Mental Disorders (PRIME-MD) [39]	X			X
Positive and Negative Affect Scale (PANAS) [40]	X	X	X	X
**Treatment credibility and preference**				
Therapy Evaluation Form [41]		X		
Treatment Preference Questionnaire		X		

### Sample size and power estimation

Input data to estimate sample size needed for adequate power came from pilot data from individuals with co-morbid chronic pain and obesity enrolled in standard care, healthy lifestyle, or intensive weight loss interventions [42] and published results from a study of weight loss intervention for older obese adults with knee osteoarthritis [43]. These data informed the following assumptions: (a) SCW is associated with an 11 pound weight loss, SCP is associated with a 1 pound weight loss, and STOP is associated with a 17 pound weight loss; (b) the within-person correlation of weight over measurements is 0.96 and the between-person standard deviation is 10 pounds; (c) SCP is associated with a 2-unit reduction in pain level (on a 10-point scale), SCW is associated with no reduction in pain level, and STOP is associated with a 4-unit reduction in pain level; (d) the within-person correlation of pain over measurements is 0.89 and the between-person standard deviation is 1.6 units; and (e) the correlation between pain level and pounds lost is 0.34. Additional assumptions included 85% retention rate over 12 months, maintenance in outcomes from 3- to 12-month follow-up, and .05 Type I error rate. Power and sample size estimates were calculated with the GLIMMPSE 2.0 online tool through Sample Size Shop’s tools for multilevel and longitudinal data [44]. A sample size of 120 participants will provide power in excess of 80% to detect between-group differences and within-person change over time while allowing for study attrition.

### Quantitative data analysis

Longitudinal outcomes will be analyzed on an intent to treat basis, including every subject who is randomized regardless of protocol adherence, study completion, or missing data. Generally, our approach will be to use mixed-effects regression models (MRMs) implemented via SAS PROC MIXED. These models do not place restrictions on the number of observations per individual; therefore, no participants will be excluded from the analysis due to missing data. In addition to using all available data, mixed models are appropriate for this study because they model individual change across repeated measures, fixed effects of baseline characteristics and treatment group, and interactions between fixed effects and continuous time [45].

For each outcome (pain intensity, weight), standard care serves as the control. For pain outcomes, STOP versus SCP and SCW versus SCP contrasts will be estimated; for weight outcomes, STOP versus SCW and SCP versus SCW contrasts will be estimated. Following the recommendations of Fitzmaurice and colleagues [46], we will model our primary outcomes at all timepoints (baseline, 3-month follow-up, 6-month follow-up, and 12-month follow-up), include both linear and quadratic effects of time and group by time interactions, control for baseline weight and baseline pain intensity, and assume the group means and variances are equal at baseline because subjects are randomized to group. Our primary tests will be for the group by time interaction terms which will indicate the extent to which treatment groups experience change in weight and pain intensity following baseline and the degree to which any observed differences are maintained over time.

### Qualitative data collection

Following the completion of treatment, all participants will be asked about their willingness to participate in a one-on-one interview about their treatment experience. To reduce bias, purposeful sampling will be employed by inviting a sample of representative participants from all three intervention groups including participants who completed and dropped out of treatment. This sampling strategy will insure that participants vary with respect to age, gender, treatment outcome, and study completion [47].

Willing participants will be interviewed by a trained member of the research staff who was not involved in leading the intervention or study assessments. Interviews are expected to last between 30 and 60 minutes. Interviewers will use a semi-structured discussion guide and follow a funnel structure progressing from broader, open-ended questions to more specific probes to clarify issues as needed. This approach will allow for flexible, thorough, and detailed data collection.

The interview protocol begins with questions designed to build rapport with participants (see Table 3 for interview protocol). Next, a series of questions elicit participants’ feedback regarding their direct experiences with the program. During the course of the interview, questions may be re-worded, re-ordered, and/or clarified to further investigate topics as introduced by the respondent. All interviews will be audio recorded and transcribed verbatim. Study participants will be invited for an interview until theoretical saturation is reached, which is the point in analysis and data gathering where all categories are well developed in terms of properties, dimensions, and variation and no new themes have been identified [48].

**Table 3:** Qualitative interview questions

**Type**	**Question**
Rapport, priorities, preference	1. Why were you interested in participating in this study?
	2. Why did you decide to participate after you learned about the study details?
	3. What were your thoughts when you found out you were randomized to [pain, weight loss, integrated intervention]?
	4. How did your view of the intervention change as you progressed through the study?
Experience	5. Describe for me the most important or useful ways to manage [weight loss, pain, both] that you learned from your sessions?
	6. What were some things covered in the sessions that were not useful for you?
	7. What do you do differently now based on what you’ve learned during treatment?
	8. How did you feel about the weekly homework assignments?
	9. How will you continue to use the skills you learned?
Experience, reflection, priorities	10. Why do you think you had the outcome you did in treatment [lose weight, experience less pain, both, neither]?
	11. What is the most important thing healthcare providers need to know about treating someone with comorbid pain and overweight?
	12. What do you think are the most important outcomes for treatment? For you, what would define a ‘good outcome’ to a treatment such as this?

### Qualitative data analysis

Once transcribed, interview text will then be verified for content accuracy by the lead investigator and two research assistants. The constant comparative method [49] will be used to analyze the data. Using this iterative approach, a pair of research assistants who did not participate in data collection, along with study staff who conducted interviews, will independently read the transcripts. They will then develop an initial list of themes from this data. Transcripts will be coded with relevant text identified for each code. Themes will be revised and refined through a second reading of study transcripts and subthemes will be created [50].

## Discussion

The proposed research is significant in that it will evaluate best-practices for behavioral management of a highly prevalent co-morbidity that has sub-optimal outcomes with currently-available treatments. The strength of this approach is its pragmatic focus. The study intervention builds upon treatments that already have demonstrated efficacy to improve pain and weight outcomes separately and are already in use in clinical practice. The current study examines these standard care behavioral approaches and compares them to an integrated approach that utilizes these evidence-based tactics in a novel way. Therefore, should an integrated treatment be found efficacious in achieving the dual outcomes of pain management and weight loss, such an approach could be integrated into clinical practice with minimal additional cost or training.

While the study will examine alternatives for treatment of pain and obesity in a setting with relevance to ‘real world’ practice, an additional advantage of this study is that the patient population will also more closely mirror those seen in clinical practice. Therefore, findings should be readily generalizable. Currently-available evidence examining treatment approaches for co-morbid chronic pain and obesity includes randomized clinical trials with very specific inclusion/exclusion criteria [16,26]. The planned intervention evaluates a treatment approach adoptable for a wider variety of pain conditions and for patients that have a number of other concurrent health issues that likely would render them excluded from most clinical trials. Thus, the patient population in STOP will closely mirror individuals who are likely to be seen in practice care settings. Findings generated from this study will have particular relevance in understanding how evidence-based approaches may perform with such populations in everyday clinical environments.

Finally, this study is innovative in that it is applying a mixed-methods approach to identify processes of change, treatment approaches, and clinical outcomes most relevant to patients. We aim to obtain potential explanations for any observed treatment effect using not only quantitative measures (e.g., Therapy Evaluation Form, Stages of Change) but also individual interviews. Qualitative research techniques such as those included here offer a comprehensive yet flexible approach particularly suited to understanding the individual’s subjective experience. Information gathered via participant interviews will offer complimentary, detailed information to aid in our understanding of treatment-promoted change in weight and/or pain outcomes. Examining how individuals with obesity and chronic pain understand and experience treatment is important to implementing more effective interventions. Indeed, such insights may provide a foundation to improve treatment implementation which will further aid in developing testable hypotheses about mechanisms of effect.

As with any intervention trial, this study has several limitations. Most significantly, while pragmatically-focused studies such as this one offer high external validity, this likely comes at a cost to internal validity. It may be difficult to identify which specific components of treatment are most actively beneficial. Additionally, while all three arms are explicitly specific in their focus (pain, weight, integrated), there is a certain degree of overlap in treatment topics across all three interventions. While we have made every attempt to adequately power the trial, it is not clear whether this overlap is significant enough to render treatment effects across the three interventions clinically and/or statistically non-significant.

In sum, the STOP study represents an important next step in understanding how to treat a common and costly co-morbidity. The trial will answer the questions of how to provide optimal care to simultaneously achieve improved pain management and weight loss. Of significant importance is the potential for the findings from this trial to be quickly implemented in existing care structures to better meet the needs of individuals with both obesity and chronic pain.

## Abbreviations

STOP: Simultaneously Treating Obesity and Pain; SCW: Standard Care Weight; SCP: Standard Care Pain; BMI: Body Mass Index; IRB: Institutional Review Board.

## Competing interests

The authors declare they have no competing interests.

## Authors' contributions

EAJ, the principal investigator, was responsible for study conceptualization and design. EAJ, MF, CH, and BH collaborated on operationalizing and delivering the intervention as described. EAJ, JMS, MF, CH, BH, and MLR participated in developing the analytic and data management plan. EAJ, MF, CH, MLR, and JMS drafted the manuscript. All authors read and approved the final manuscript.

## Pre-publication history

The pre-publication history for this paper can be accessed here:

http://www.biomedcentral.com/1471-2458/14/621/prepub
